# Surgical removal of a telemetry system in a cynomolgus monkey (*Macaca fascicularis*): a 12-month observation study

**DOI:** 10.1186/s42826-021-00106-z

**Published:** 2021-10-16

**Authors:** Doo-Wan Cho, Hyoung-Yun Han, Mi-Jin Yang, Dong Ho Woo, Su-Cheol Han, Young-Su Yang

**Affiliations:** 1 Jeonbuk Branch Institute, Korea Institute of Toxicology, Jeongeup, Republic of Korea; 2 Molecular Toxicology Research Group, Korea Institute of Toxicology, Daejeon, Republic of Korea; 3 Pharmacology & Drug Abuse Research Group, Korea Institute of Toxicology, Daejeon, Republic of Korea

**Keywords:** Cynomolgus monkey, Surgery, Telemetry, Implantation, Welfare

## Abstract

**Background:**

Telemetry is a wireless implanted device that measures biological signals in conscious animals and usually requires surgery for its removal when the study is finished. After removing the device, the animals are either used for other studies or euthanatized.

**Case presentation:**

Herein, we report the case of a living cynomolgus monkey (*Macaca fascicularis*) that was used for the entire experimental period, instead of euthanasia, after surgical removal of an implanted telemetry system. Radiography was used to determine the status of the implanted telemetry, following which, a repair surgery was performed for removing the system; clinical signs were used to preserve the life of the cynomolgus monkey. Postoperative clinical signs, food consumption, hematology, and serum biochemistry were examined during the 12-month observational period. No abnormal readings or conditions were observed in the subject after implant removal.

**Conclusions:**

This study may be a useful case report for living cynomolgus monkeys in telemetry implantations used throughout the study period. We suggest minimizing the suffering and improving the welfare of these animals.

**Supplementary Information:**

The online version contains supplementary material available at 10.1186/s42826-021-00106-z.

## Background

When evaluating a new pharmaceutical product, the essential requisite is the drug’s safety, particularly, the potential cardiovascular effects in humans. As a result, safety assessments are performed following the International Council on Harmonization guidelines. Precise and detailed measurements are required, especially, of arterial pressure because of its narrow physiological safety range. The cardiovascular system is particularly vulnerable to changes depending on the animal’s environmental comfort. For this reason, implanted telemetric systems have been developed and widely used [1–3]. The telemetry systems installed have been specifically designed to allow data collection without human contact interference to ensure accurate blood pressure readings and electrocardiogram waveforms during the entire study period.

The primary types of animals used for testing with such telemetry systems are rodents, dogs and primates. Regarding the insertion and management of telemetric sensors, more attention is needed in primate species because of their smaller abdominal cavities and picking at sutured skin margins around an implant, complicating healing. The animals receiving telemetric implants recover after approximately 2–3 weeks and eventually undergo pharmacological safety tests for about two and a half years. At this point, the sensor battery is no longer effective, and the sensor is removed. Most animals are killed at the end of studies for scientific or humane reasons [4]. In addition, some countries have legal constraints that apply to their reuse. Some animals are reused or rehomed. However, there are no reports on reused animals after telemetry implant removal surgery.

In this study, we report the case of a reused animal and described its health data during a postoperative 12-month observational period.

## Case presentation

Animals were housed and maintained in the specific pathogen-free (SPF) facility at the Korea Institute of Toxicology (KIT), according to KIT Animal Care and Use Committee Guidelines. All monkeys were kept in an indoor individual cage and was fed commercial monkey chow (Certified Primate Diet #5048, PMI Nutrition International, Inc., USA) supplemented daily with various fruits, and supplied water ad libitum. The temperature, humidity, and light cycle were automatically controlled (20–29 °C, relative humidity 40–70%, 12 h light/dark cycle with 300–700 lx). This study was conducted with the approval of the Animal Experiment Ethics Committee (KIT IACUC number: 1609-0325).

**Fig. 1:**
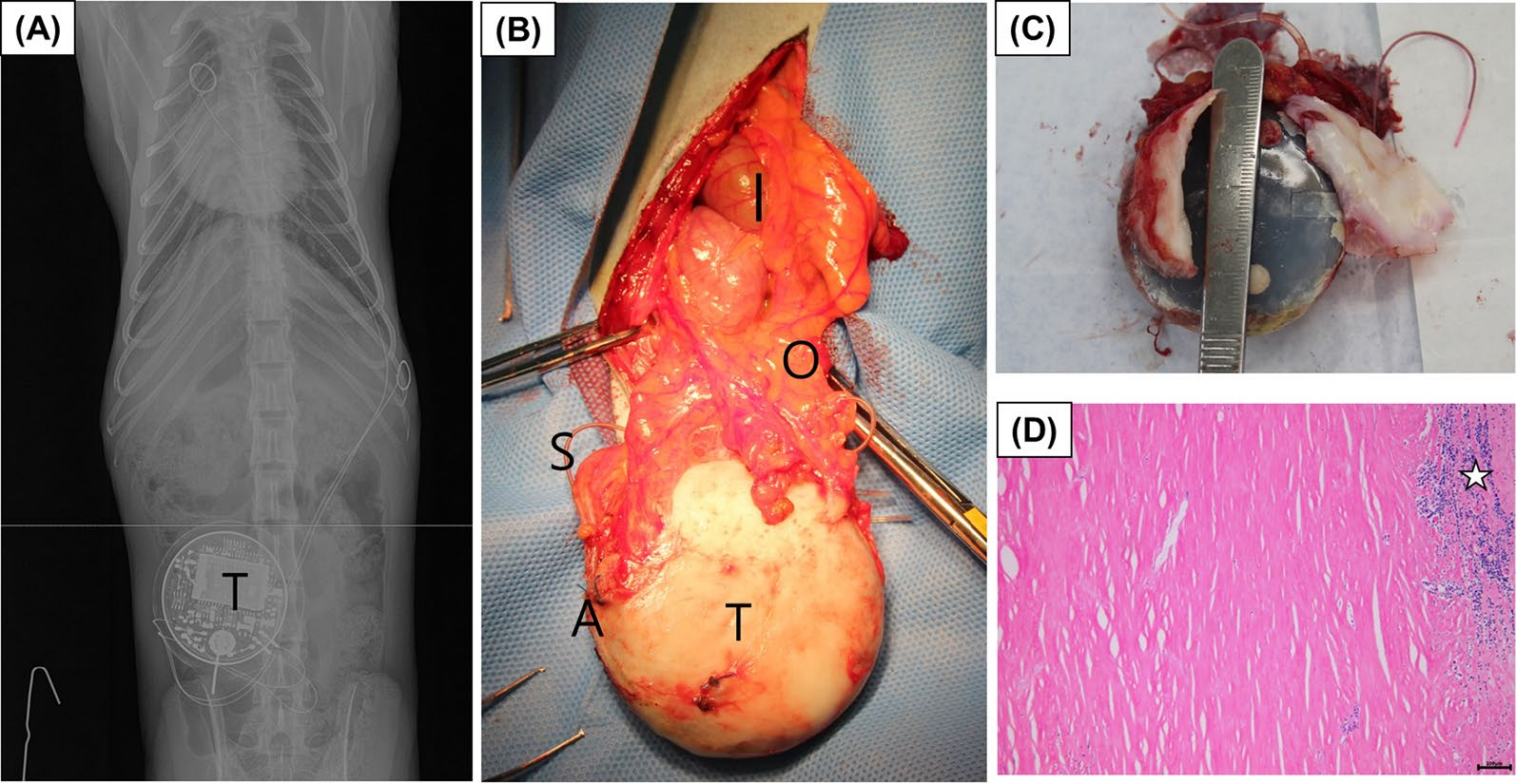
Images for experimental procedures. X-ray image was before the removal surgery (**A**). A transmitter was removed by surgical method (**B**) and it was capsuled by fibrous substance (**C**) that was composed with collagen fiber and slight lymphocyte infiltrated (white star) in microscopic examination (**D**) (Scale bar = 100 µm). Alphabets in image were Intestine (I), Omentum (O), Sensor (S), Transmitter (T) and Attachment site in abdominal wall (A)

Six male cynomolgus monkeys (*Macaca fascicularis*), also known as the long-tailed macaque, were used in the tests and the telemetry system that had been installed for 28 months was removed in one monkey. This was because this animal was rarely used in toxicological studies than the others. On the day preceding the implant removal surgery, the subject was fasted and general anesthesia was induced via intramuscular ketamine injection. A radiography (Fig. 1A) was performed after anesthesia induction and the intricacies of the sensor location and installation were verified, and the removal procedure was initiated. The animal was intubated and artificially ventilated using a mixture of oxygen and volatile anesthetic (isoflurane 1–2%), throughout the operation. The animal’s mid-abdominal region was shaved, depilated, disinfected by using a 2-stage surgical scrub with 70% ethanol and povidone-iodine, and covered with a sterile surgical drape. An incision was made along the linea alba using an electrosurgical scalpel, which minimized bleeding. Upon opening the abdominal cavity, the transmitter was encapsulated by tissue, surrounded by attached connective, omentum, and fatty tissues (Fig. 1B). The transmitter was extracted by carefully removing the sensor anchor as well as the surrounding soft tissue and sensor lines fixed to the peritoneum. The abdominal cavity was cleaned twice with sterile physiological saline. Once the rinsing was complete, the abdominal wall was closed in layers using the simple interrupted suture pattern and 4–0 absorbable suture material, and the subcutaneous tissue closed using a continuous suture pattern and 4–0 Vicryl® Ethicon absorbable suture material. The skin margin was closed using simple interrupted suture pattern and 4–0 non-absorbent suture material. Antibiotics (cefazolin sodium 20 mg/kg, b.i.d., five days; Chong Kun Dang Pharm) and analgesics (ketoprofen; 2 mg/kg, s.i.d., five days; UNIBIOTECH) were administered after the surgery, and no particular abnormalities were observed, based on the results of the clinical observation, hematological and biochemical tests conducted postoperatively (Additional file 1: Table S1).

Postoperative radiography was performed, and microscopic examination of the tissue wrapping the transmitter showed that some lymphocytes were present in the excised fibrous tissue (Fig. 1D). Twelve months after the removal, the animal showed no unusual findings in clinical signs, food consumption, and clinical chemistry examination results (Table 1).

**Table 1:** Clinical chemistry results were for implant surgery (pre and post data) and removal surgery (pre and post data)

Parameters	Implant surgery		Removal surgery	
	Pre (Day -7)	Post (Day 14)	Pre (Day -30)	Post (12 months later)
GLU (mg/dL)	96.7	69.4	62.8	75.8
BUN (mg/dL)	19.3	18.9	19.9	16.2
CREA (mg/dL)	0.83	0.86	0.74	0.75
TP (g/dL)	7.27	8.31	7.17	7.68
ALB (g/dL)	4.38	4.69	4.14	4.54
A/G (ratio)	1.52	1.3	1.37	1.45
AST (IU/L)	36.9	47.8	25.9	36.5
ALT (IU/L)	31.8	37.6	24.5	24.2
TBIL (mg/dL)	0.303	0.363	0.238	0.268
GGT (IU/L)	87.72	80.04	66.1	56.89
RBC (× 10^6/µL)	6.33	6.41	6.25	6.3
WBC (× 10^3^/µL)	12.03	18.06	11.55	8.66
NEU (× 10^3^/µL)	5.65	12.85	6.31	3.17

## Discussion and conclusions

The animal that had removal surgery was exposed to new materials in a single low volume, and no particular unusual findings were observed in the general symptoms and feed collection observations prior to the sensor removal operation. The fibrous substance observed in the animal may be a protective mechanism elicited by the body against external materials [5]. This was considered as a complex wound healing response to the presence of biomedical device with diverse cell signaling, followed by migration of fibroblasts to the implant surface. Eventually, it is walling off of the implant in a collagen capsule. Similar results has been reported in experiment with implanting other biomedical devices into the experimental animals [6].

After removing the transmitter, we regularly examined clinical signs, food consumption, body weight, and clinical pathology, and no uneventful findings were noted. Microbiological tests were periodically conducted on the monkey, such as for *Salmonella, Shigella,* and *Yersinia*, and no parasites were isolated from fecal samples. During social housing with other animals, the animal played well and groomed each other. One year later, the animal remained healthy without any abnormal health conditions when compared with aged-matched surgery-naïve animals. The animal is currently being used for non-invasive laboratory procedures (mating and semen extraction).

Recently, various sensors or devices used to obtain physical data, cardiovascular effects, and blood glucose measurements have been developed and inserted into animal bodies for accurate and continuous testing results from a conscious, movement-free situation [2, 7, 8]. Most animals used in such studies are euthanized at the end of the study. However, we need to consider whether euthanasia is the only effective and convenient method for the experimenter. When making the final judgment, we should take into account the scientific and ethical problems of reusing animals and obtain the opinions of the Institutional Animal Care and Use Committees (IACUC) committee members and make a final decision [7, 8].

In this study, we present the long-term animal health data after surgical removal of a telemetry implant. However, this is a single case report with a monkey, more reports or clinical trials with other animals study are needed. We hope that this study may help to determine the fate of animals used in telemetry studies.

## Supplementary Information

Additional file 1: Table S1. Clinical chemistry results for implant surgery (pre and post data) in five animals. Telemetry device was implanted in six animals and removal surgery was conducted in only one animal. Other five animals were euthanized in accordance with the study design.

## Data Availability

The authors confirm that the data supporting the findings of this study are available within the article [and/or] its supplementary materials.

## Abbreviations

ALB: Albumin
ALT: Alanine aminotransferase
AST: Aspartate aminotransferase
A/G: Albumin/globulin ratio
BUN: Blood urea nitrogen
CREA: Creatinine
GGT: Gamma glutamyl transpeptidase
GLU: Glucose
NEU: Neutrophil
RBC: Red blood cell
TBIL: Total bilirubin
TP: Total protein
WBC: White blood cell
