# Supplementary material for: Surgical removal of a telemetry system in a cynomolgus monkey (Macaca fascicularis): a 12-month observation study
Source: Lab Anim Res. 2021 Oct 16;37:29. doi: 10.1186/s42826-021-00106-z (PMC8520245; doi:10.1186/s42826-021-00106-z)
Supplement: Supplementary file 1 — Clinical chemistry results for implant surgery (pre and post data) in five animals. Telemetry device was implanted in six animals and removal surgery was conducted in only one animal. Other five animals were euthanized in accordance with the study design. [file 42826_2021_106_MOESM1_ESM.docx]

**Supplementary Table S1.** Clinical chemistry results for implant surgery (pre and post data) in five animals. Telemetry device was implanted in six animals and removal surgery was conducted in only one animal. Other five animals were euthanized in accordance with the study design.

| Parameters | Implant surgery (n =5) | | Removal surgery | |
| --- | --- | --- | --- | --- |
|  | Pre (Day -7) | Post (Day 7) | Pre (Day -30) | Post (12 months later) |
| GLU (mg/dL) | 48.2±23.40 | 76.5±32.42 | - | - |
| BUN (mg/dL) | 18.9±8.34 | 20.4±8.37 | - | - |
| CREA (mg/dL) | 0.77±0.380 | 0.76±0.353 | - | - |
| TP (g/dL) | 6.80±3.502 | 5.81±3.063 | - | - |
| ALB (g/dL) | 3.85±2.030 | 3.58±1.926 | - | - |
| A/G (ratio) | 1.1±0.52 | 1.34±0.659 | - | - |
| AST (IU/L) | 62.7±29.17 | 36.6±14.13 | - | - |
| ALT (IU/L) | 37.1±17.49 | 29.8±8.30 | - | - |
| TBIL (mg/dL) | 0.281±0.1336 | 0.214±0.1154 | - | - |
| GGT (IU/L) | 74.28±33.848 | 89.48±39.088 | - | - |
| RBC (x10^6/µL) | 4.74±2.532 | 4.84±2.571 | - | - |
| WBC (x10³/µL) | 7.68±3.654 | 11.03±5.535 | - | - |
| NEU (x10³/µL) | 4.12±1.891 | 7.83±3.961 | - | - |
